# Dynamic allostery in thrombin—a review

**DOI:** 10.3389/fmolb.2023.1200465

**Published:** 2023-06-29

**Authors:** Elizabeth A. Komives

**Affiliations:** Department of Chemistry and Biochemistry, University of California San Diego, La Jolla, CA, United States

**Keywords:** serine protease, thrombomodulin, isothermal titration calorimetry, HDX-MS (hydrogen/deuterium exchange-mass spectrometry), NMR relaxation dispersion

## Abstract

Thrombin is a serine protease that catalyzes a large number of different reactions including proteolytic cleave of fibrinogen to make the fibrin clot (procoagulant activity), of the protease activated receptors (for cell signaling) and of protein C generating activated protein C (anticoagulant activity). Thrombin has an effector binding site called the anion binding exosite 1 that is allosterically coupled to the active site. In this review, we survey results from thermodynamic characterization of the allosteric coupling as well as hydrogen-deuterium exchange mass spectrometry to reveal which parts of the thrombin structure are changed upon effector binding and/or mutagenesis, and finally NMR spectroscopy to characterize the different timescales of motions elicited by the effectors. We also relate the experimental work to computational network analysis of the thrombin-thrombomodulin complex.

## 1 Introduction

**FIGURE 1:**
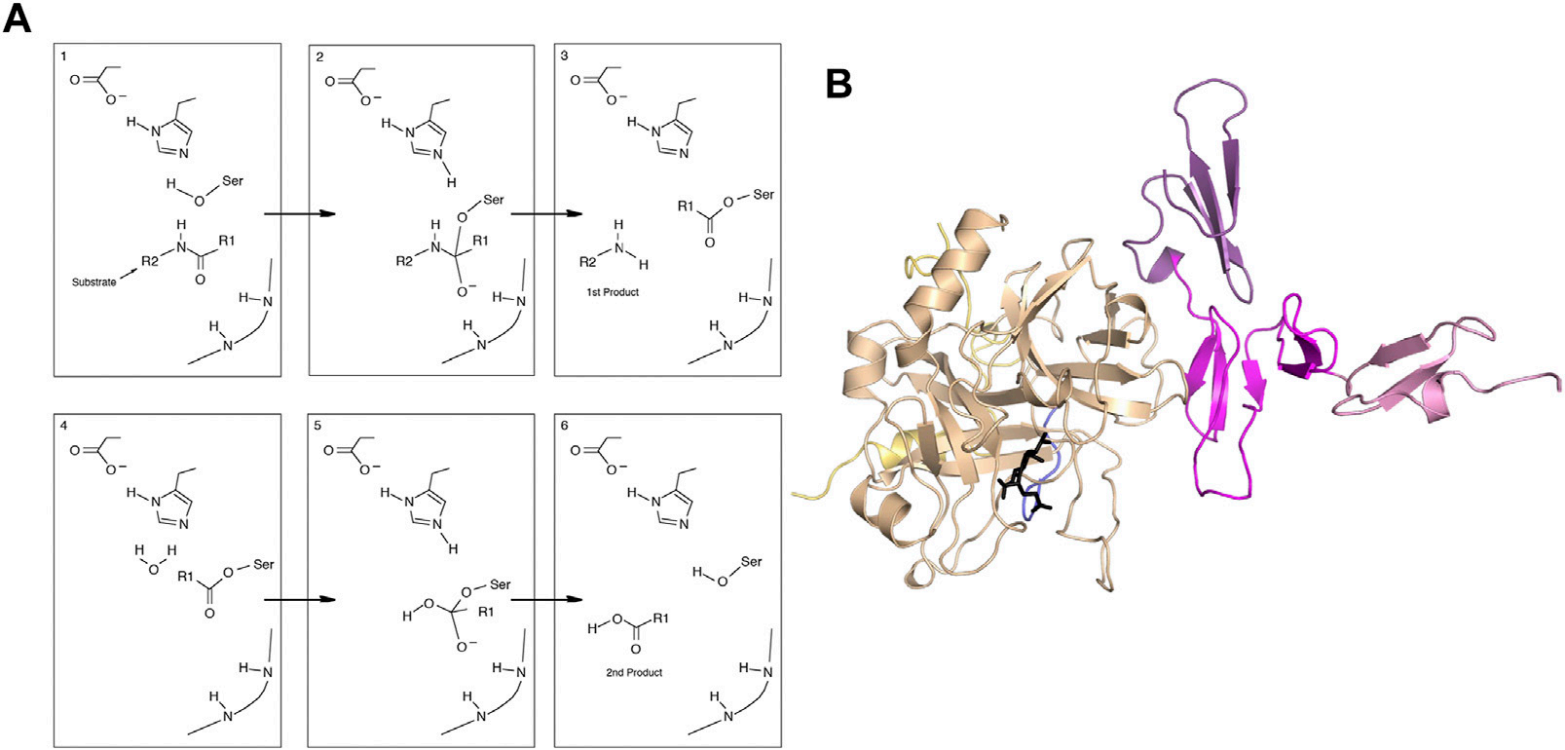
**(A)** General mechanism of serine proteases. **(B)** Structure of the thrombin-thrombomodulin complex (PDB 1DX5) showing thrombin (sand), with the light chain (yellow) and the new N-terminus (blue). Thrombomodulin is colored by EGF-like domain with EGF4 in pink, EGF5 in magenta and EGF6 in purple.

Thrombin is one of the best-studied serine proteases. The mechanism of serine proteases is multistep and involves covalent intermediates (Figure 1A). Depending on the substrate, reactions are slow compared to simpler enzymes (kcat for cleavage of fibrinogen by thrombin is 84 s-1 (Higgins et al., 1983) whereas the kcat for adenylate kinase is 190 s-1 (Kerns et al., 2015) and kcat for triosephosphate isomerase is 4,300 s-1 (Straus et al., 1985). Allostery usually implies alteration in binding affinity by binding of an effector molecule at a second site. The case of thrombin is very interesting because it has a large number of different substrates and effectors change the activity of the enzyme towards one or another. Na + binding, for example, favors the cleavage of fibrinogen to form the fibrin clot (procoagulant activity) whereas thrombomodulin (TM) binding favors the proteolytic activation of protein C to form activated protein C (anticoagulant activity). Allostery also usually involves a structural change that can be detected by crystallography, however the structure of thrombin does not seem in many cases to be reflective of its activity.

Observations that thrombin may be an allosteric protein began to appear in the 1980s, even before the first crystal structures appeared. In 1981, Esmon discovered a cofactor, later named thrombomodulin (TM), that “switched” the activity of thrombin from pro-coagulant to anti-coagulant (Esmon and Owen, 1981). This “switch” behavior fascinated biophysicists who began to examine the conformational changes induced by TM once a smaller functional fragment of TM was isolated (Stearns et al., 1989). Using biophysical probes such as spin labels (Musci et al., 1988) and fluorophoric (Ye et al., 1991), biophysicists demonstrated that TM-binding caused detectable alterations in the active site of thrombin. As with other allosteric systems, site-directed mutagenesis of key residues could partially shift the equilibrium of states to favor one or the other as demonstrated by mutation of a key TM-binding residue Glu192 to Gln (Le Bonniec and Esmon, 1991). Later structural studies revealed conformational changes caused by this mutation (van de Locht et al., 1997).

Binding of adenosine nucleotides also modulated the activity of thrombin, and a full allosteric linkage analysis demonstrated these molecules were allosteric effectors (De Cristofaro et al., 1990). In 1992, Di Cera’s group discovered that thrombin activity is modulated by Na + binding (Wells and Di Cera, 1992), a phenomenon that had previously been reported for other blood coagulation serine proteases (Steiner and Castellino, 1985). Here again, once structural studies emerged (Zhang and Tulinsky, 1997), it was possible to find residues such as Trp 60D, far from the Na + binding site, which shifted the equilibrium of states (Guinto and Di Cera, 1997). Furthermore, despite the very small interaction surface expected for Na + binding, a large heat capacity change was observed upon Na + binding hinting that Na + binding may be a dynamic allosteric event. Most interestingly, the Na + binding and TM binding appeared to be allosterically coupled (Dang et al., 1995). That meant that thrombin has at least 3 binding sites that might be coupled–the active site, the TM-binding site and the Na + -binding site.

## 2 Thermodynamic signatures of thrombin allostery

**FIGURE 2:**
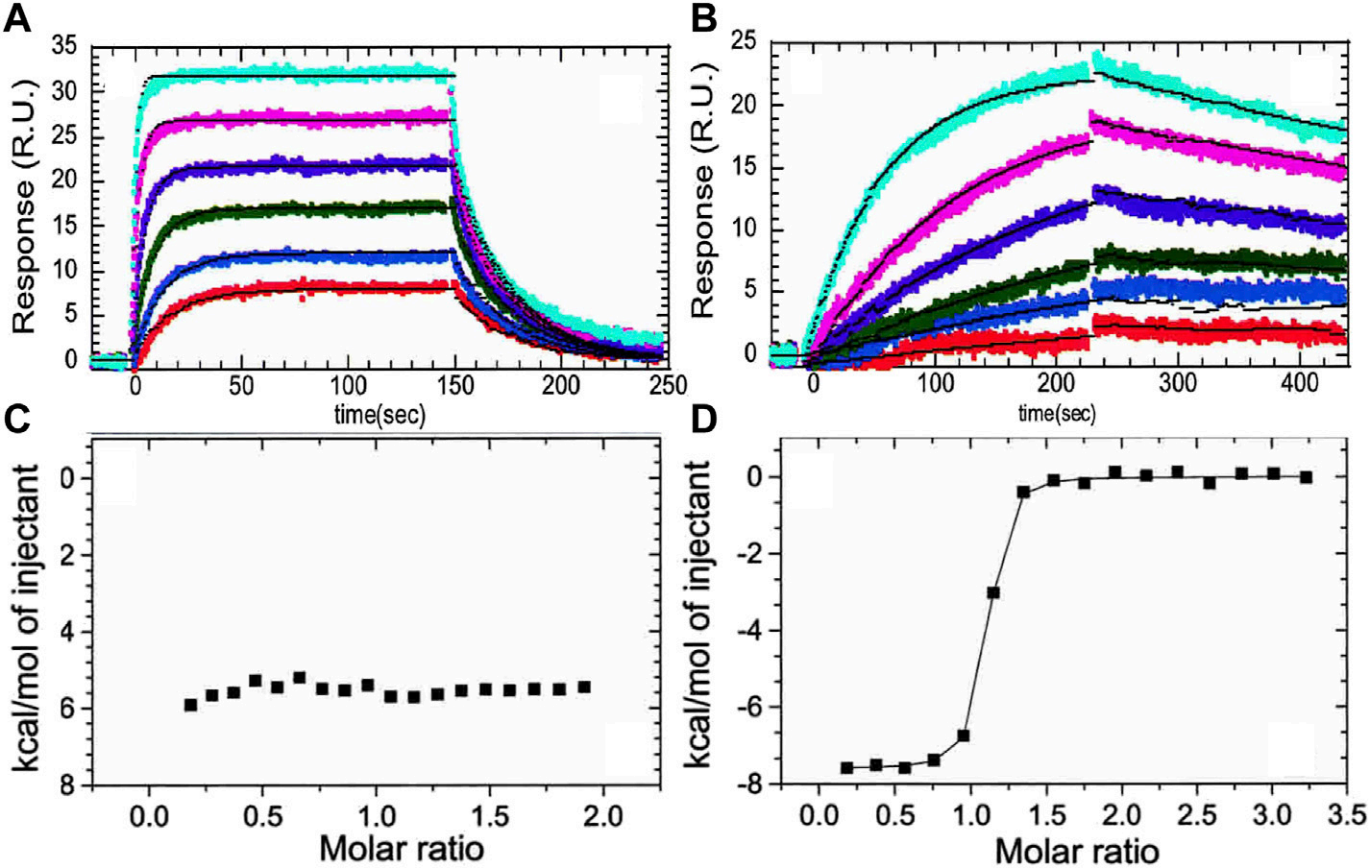
**(A)** Surface plasmon resonance traces of thrombin (0.78, 1.56, 3.125, 6.25, 12.5, 25 nM) binding to TM immobilized by way of N-terminal biotin. **(B)** The same concentrations of thrombin binding to the monoclonal antibody (AHT-5020 from Prolytix, Inc). **(C)** Isothermal titration calorimetry trace of TM (70 µM in the syringe) binding to thrombin (6 µM in the cell). **(D)** Isothermal titration calorimetry trace of the monoclonal antibody (70 µM in the syringe) binding to thrombin (6 µM in the cell). The data in this figure are from Baerga-Ortiz et al. (2004).

The crystal structure of the thrombin-TM complex revealed that TM only contacts thrombin via its fifth EGF-like domain despite the fact that the fourth EGF-like domain is required to “switch” the catalytic activity of thrombin (Fuentes-Prior et al., 2000) (Figure 1B). Our group attempted to measure the thrombin-TM binding by surface plasmon resonance and isothermal titration calorimetry with the goal of determining the coupling between Na + -binding and TM binding. By SPR, we could measure a Kd of 4 nM for the smallest active fragment of TM comprised of the 4th, 5th, and 6th EGF-like domains (Baerga-Ortiz et al., 2000) (Figure 2A). The interaction was strongly electrostatically-driven as might be expected from the highly positively-charged TM binding site on thrombin and the fact that TM is highly negatively charged. Having demonstrated that we could detect binding, we attempted to measure the thermodynamics of binding by isothermal titration calorimetry (ITC). We could not measure any binding of TM to thrombin by ITC (Baerga-Ortiz et al., 2004) (Figure 2C). As a control, we purchased a monoclonal antibody that was reported to bind in the TM binding site. By SPR, the antibody bound with the same binding affinity as TM, but the association and dissociation rates were much slower than the rates observed for TM binding (Figure 2B). The antibody binding was driven by a favorable ΔH as detected by ITC (Figure 2D) (Baerga-Ortiz et al., 2004). We surmised that the two interactions must have different thermodynamic driving forces with the thrombin-TM binding being entropically-driven. This was the first hint that thrombin may undergo dynamic allostery.

It is important to point out that the TM binding site on thrombin (Figure 1B) is called anion binding exosite 1 (ABE1). Fibrinogen interacts with thrombin at both the active site AND ABE1 (Meh et al., 1996). The inhibitor isolated from leeches, hirudin, also interacts at both the active site AND ABE1 (Grütter et al., 1990). We initially developed the use of amide hydrogen-deuterium exchange mass spectrometry (HDX-MS) for detecting protein-protein interfaces (Mandell et al., 1998). The results for the thrombin-TM interaction were puzzling because we not only detected the binding site, which was already pretty well known, but we detected changes throughout thrombin that were unexpected. Most of the active site loops showed subtle decreases in exchange when TM bound. To further probe interfacial binding, we performed the H/D exchange at different pHs. We surmised that if TM was bound to thrombin, then the solvent exclusion at the interface would be pH independent whereas if TM were causing changes in loop dynamics, the H/D exchange in the loops would continue to show a pH dependence. Indeed, two segments of thrombin became completely solvent-inaccessible, as evidenced by the pH insensitivity of the amide H/D exchange rates. These segments formed part of anion-binding exosite I and contained the residues for which alanine substitution abolished TM binding (Mandell et al., 2001). The decreased exchange in several active site loops remained pH sensitive indicating that these changes were not due to solvent accessibility changes due to direct binding. These results suggested an allosteric connection between ABE1 and the active site.

**FIGURE 3:**
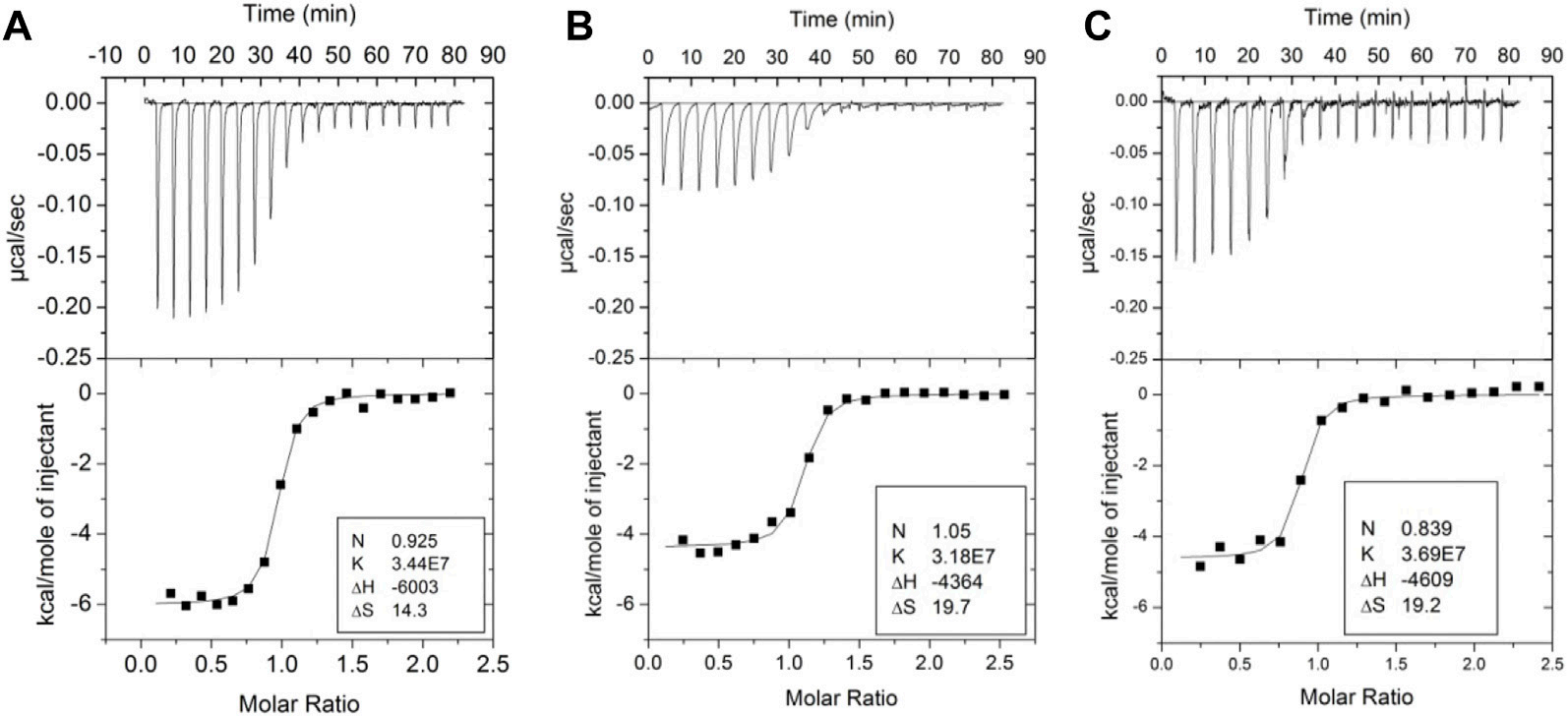
Isothermal titration calorimetry traces of various ligands binding to thrombin. **(A)** DAPA (70 µM in the syringe) binding to thrombin (6 µM in the cell). **(B)** DAPA (70 µM in the syringe) binding to the thrombin-aptamer complex (6 µM in the cell). **(C)** DAPA (70 µM in the syringe) binding to the thrombin-TM complex (6 µM in the cell). These data are from Trueheit et al. (2011).

Several groups have demonstrated the thermodynamic linkage between ABE1 and the thrombin active site using ITC. Binding of the lectin, bothrojaracin at ABE1 was shown to cause changes in the thrombin active site (Monteiro et al., 1999). Krishnaswamy’s group used ITC to demonstrate thermodynamic linkage between anion-binding exosite 2, the Na (+)-binding site, and the active site arises from interconversions of thrombin between a continuum of zymogen- and proteinase-like states. We also took advantage of aptamers selected to bind to ABE1and inhibitors selected to bind to the active site to understand the thermodynamics of the allostery between these sites. Our experiments showed that the inhibitor, Dansylarginin, N-(3-ethyl-1.5-pentanediyl)amid (DAPA), bound to the active site of thrombin with a ΔH of −6 kcal/mol and this was reduced to −4.4 kcal/mol when a DNA aptamer (Wu et al., 1992) was bound to ABE1 (Figures 3A, B) (Treuheit et al., 2011). A similar change was observed with TM (EGF4-5) was bound to ABE1 prior to DAPA binding (Figure 3C). In both cases, the difference in the enthalpy change upon binding was compensated by a compensating difference in the entropy change upon binding so that the overall association constant was unchanged. Whereas allostery typically results in a change in the binding affinity at one site when the other site is occupied, the linkage between ABE1 and the thrombin active site shows enthalpy-entropy compensation indicative of dynamic allostery.

## 3 Structure/function analyses of thrombin allostery

**FIGURE 4:**
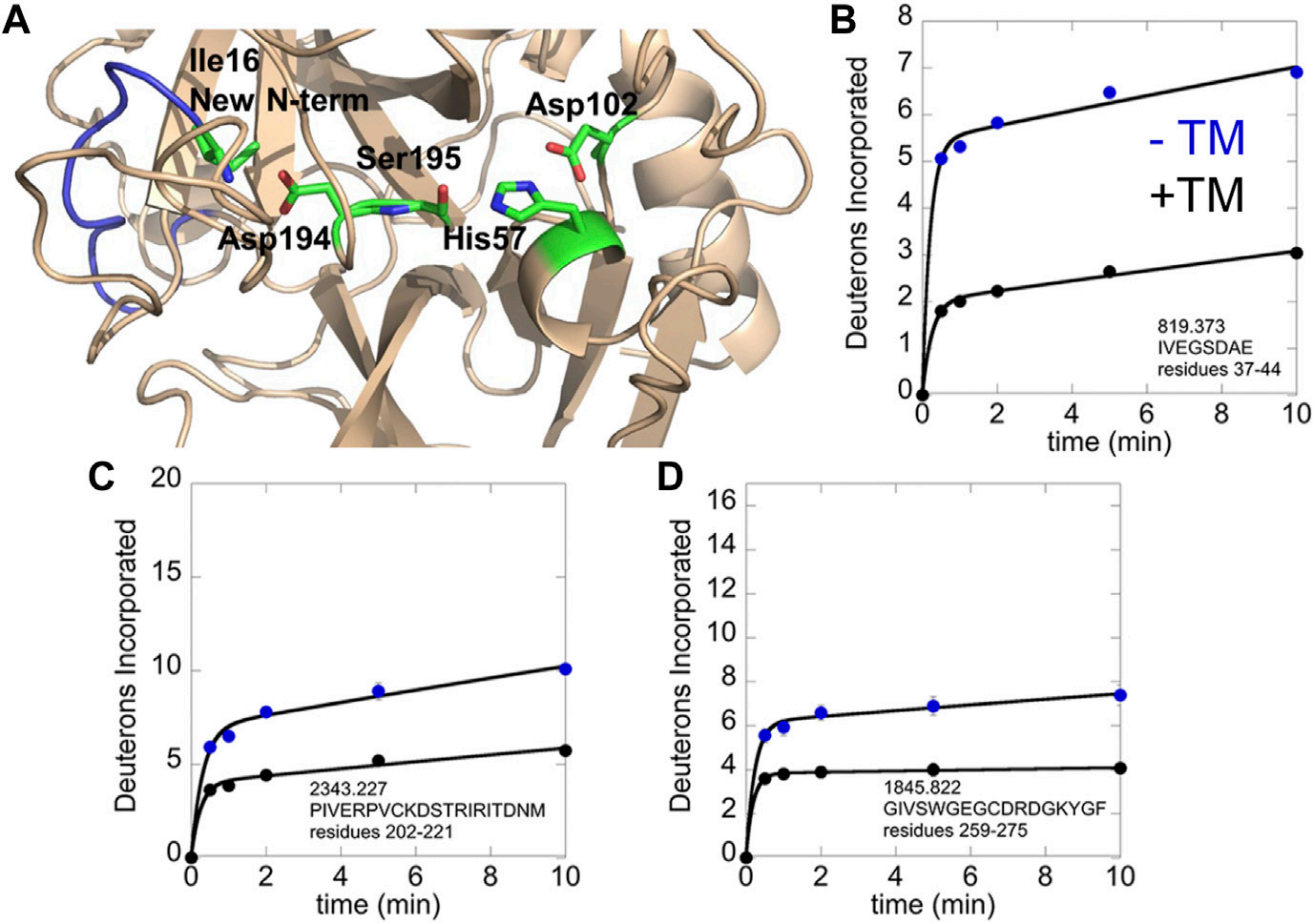
HDX-MS analysis of the thrombin-TM complex as compared to thrombin alone. **(A)** structure of thrombin showing the new N-terminus (blue) with Ile16 and the catalytic triad in sticks. **(B)** Deuterium incorporation into the new N-terminus of thrombin alone (blue) vs. in the thrombin-TM complex (black). **(C)** Deuterium incorporation into the 180 s loop of thrombin alone (blue) vs. in the thrombin-TM complex (black). **(D)** Deuterium incorporation into the Na + binding loop of thrombin alone (blue) vs. in the thrombin-TM complex (black).

We revisited the amide exchange in thrombin later when mass spectrometry instrumentation had improved resulting in near 100% sequence coverage. Using the more up-to-date HDX-MS platform, we discovered an important aspect of TM-mediated allostery that was missed due to inadequate sequence coverage in the original thrombin HDX experiments. TM markedly stabilizes the active site conformation in which the new N-terminus is docked in the isoleucine binding pocket. All serine proteases follow the same activation mechanism in which they exist in a zymogen form until proteolytic cleavage at the activation peptide. As is the case for thrombin, many serine proteases contain cysteine disulfide bonds and the cleaved polypeptide remains attached to the protease via a disulfide bond. In thrombin, cleavage is after Arg, and the new N-terminal residue is an Ile. In all crystal structures of thrombin, one can observe the new N-terminal amino group interacting with Asp 194, which is adjacent to the catalytic Ser 195 (Figure 4A). We were surprised that in thrombin that did not have an inhibitor bound, the new N-terminal region was highly exchanging (Figure 4B). TM-binding markedly stabilizes this region, even though its binding site is distant (cf Figure 1B). In addition, TM binding stabilized the regions adjacent to and including the Na + -binding site (Figures 4C, D).

Besides analyzing the thrombin-TM interaction, we also analyzed a number of thrombin mutants that had also been studied by other groups and for which crystal structures were available. Three mutants of particular interest will be discussed. The first is Glu 192 which when mutated to Gln was reported to mimic the catalytic switch induced by TM (Le Bonniec and Esmon, 1991). This mutation was later shown to alter the structure of thrombin when the active site was occupied (van de Locht et al., 1997). The mutation of Glu217 was discovered to “convert” thrombin into an anticoagulant (Gibbs et al., 1995; Cantwell and Di Cera, 2000). Glu 217 is near the Na + binding site formed by octahedral coordination to the main chain oxygens of Arg-221a and Lys-224 and four water molecules (Di Cera et al., 1995; Zhang and Tulinsky, 1997). It is also near Trp215 which was implicated in moving out of the active site when Na + binds (Wells and Di Cera, 1992). Multiple other studies have shown that the Trp215 side chain of thrombin can occlude the active site upon mutation of residues near the active site such as D102N (Gandhi et al., 2008) Δ146−149e (Bah et al., 2009), Y225P (Niu et al., 2011) and N143P (Niu et al., 2009). Trp215 is mutated in the well-studied anticoagulant W215A/E217A mutant that is referred to as “WE thrombin” (Gruber et al., 2002). The anticoagulant nature of these mutant thrombins is linked to the structure of the Na + binding site. The structure of inhibitor and Na + -free thrombin shows that both Trp-215 and Arg-221 occlude the active site and the primary specificity pocket. The authors surmised that in the absence of Na+, thrombin may assume an inactive conformation with an occluded active site (Pineda et al., 2006).

**FIGURE 5:**
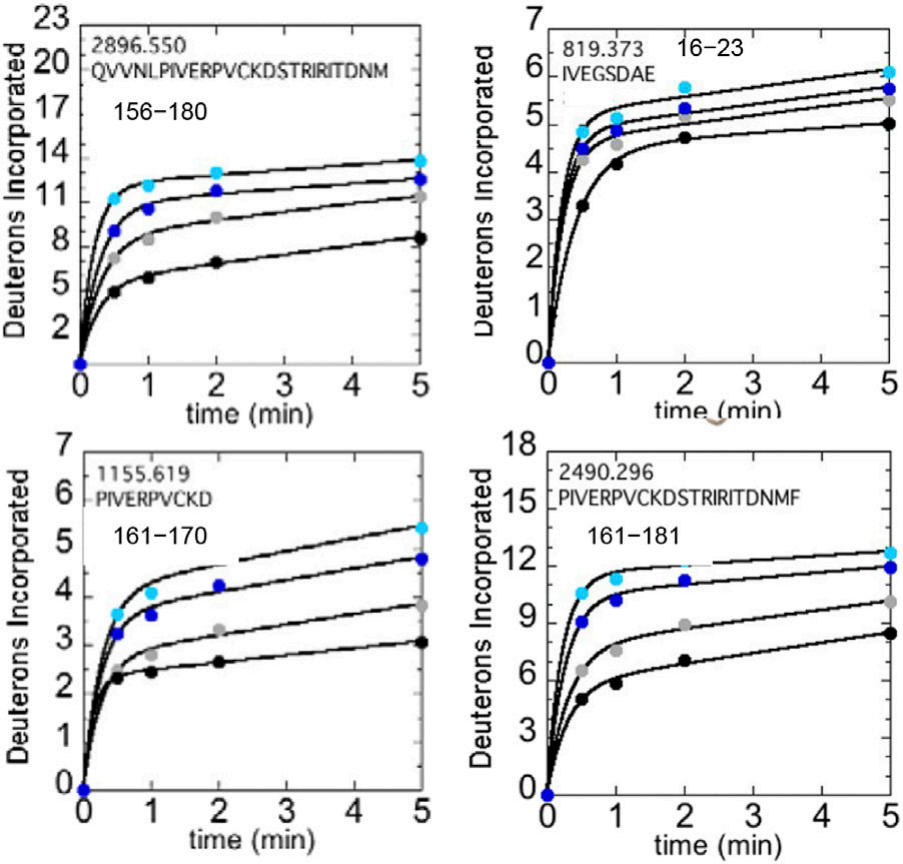
Structure of WT thrombin (PDB 1PPB) highlighting 156–181 (residues 197–222; green). The side chains of Trp215 (blue) and the catalytic triad (black) are shown as sticks. Deuterium incorporation over 5 min into residues 156–180, 161–170, and 161–181 is shown for WT thrombin at 100 mM NaCl (gray) and 300 mM NaCl (black), and for the W215A mutant at 100 mM NaCl (cyan) and 300 mM NaCl (blue).

We used HDX-MS and accelerated molecular dynamics (AMD) to study the backbone dynamics of the W215A mutant thrombin and compared it to wild type thrombin (Peacock et al., 2018). Due to the apparent π-stacking interaction between Trp215 and Phe227, we included mutants of Phe227 in our study as well. W215A mutant has markedly increased dynamics in the 170sCT loop, the 220s loop, and the N-terminus of the heavy chain, and its dynamics are less sensitive to NaCl concentration (Figure 5). Investigation of the π-stacking interaction between Phe227 and Trp215 revealed that this interaction is critical for ordering the 170s loop, but Trp215 alone is responsible for long-range allostery that impacts the 220s loop and the N-terminus of the heavy chain.

## 4 Computational network analysis of thrombin allostery

Accelerated molecular dynamics has the challenge that it is not trivial to determine how much to accelerate (i.e., how much to flatten the energy landscape) to explore realistic motions of proteins on longer timescales. We demonstrated that the AMD simulations could be calibrated by comparing experimental residual dipolar couplings with those back-calculated from the Boltzmann-reweighted AMD ensemble (Fuglestad et al., 2012). Allosteric network analysis was carried out on the ensembles obtained from AMD of thrombin alone vs. thrombin in complex with TM (Gasper et al., 2012). This analysis revealed several very interesting findings. First, thrombin alone had many small regions of uncorrelated motions whereas the motions became highly correlated in TM-bound thrombin. Even more interesting, however, was the observation that motions within the EGF-like domains of TM were coupled to motions within thrombin. This result suggested that the TM-thrombin complex behaves like a single dynamic entity to catalyze the activation of protein C.

## 5 The multiple timescales of dynamic allostery in thrombin

**FIGURE 6:**
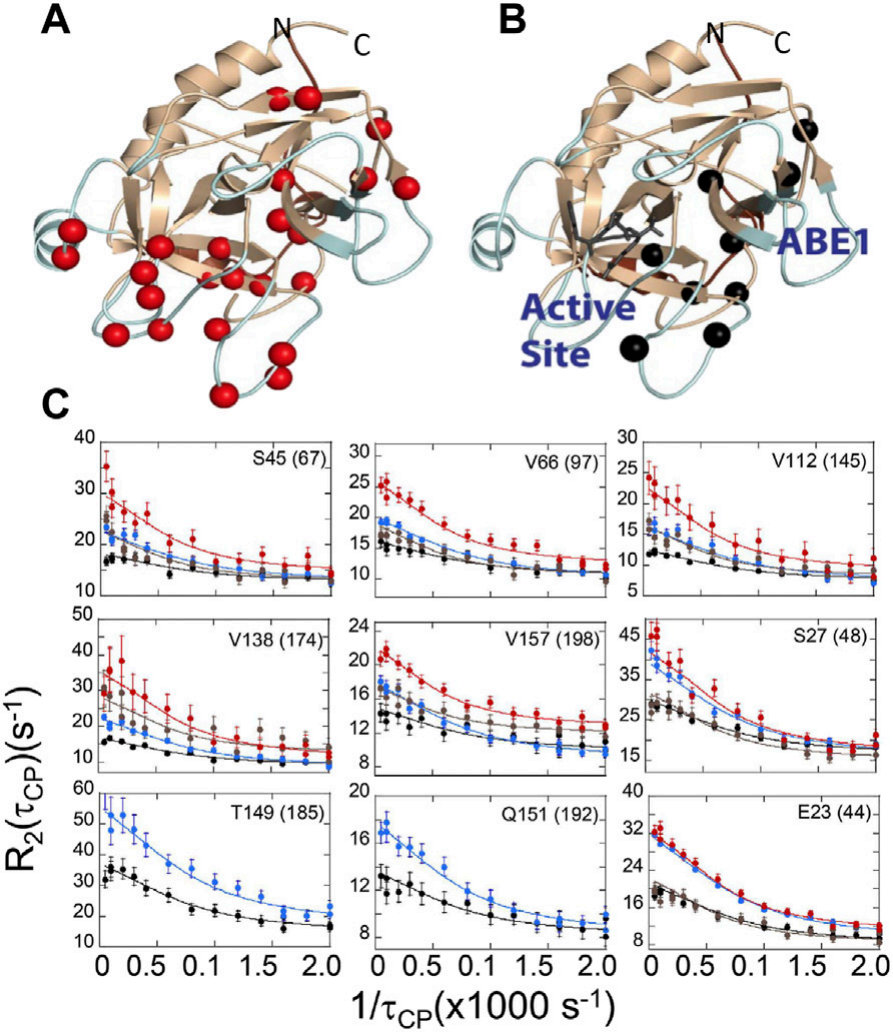
Relaxation dispersion results for **(A)** thrombin and **(B)** PPACK-inhibited thrombin. The spheres in each structure are those amide groups showing significant Rex. **(C)** Example dispersion curves and data points for apo-thrombin at 800 MHz (red) and 600 MHz (brown); and for PPACK-thrombin at 800 MHz (blue) and 600 MHz (black).

The evidence from ITC and HDX-MS showed clearly that binding of TM to thrombin or mutation of specific amino acids could markedly alter the dynamics of thrombin far from the site of perturbation. However, the timescales of the motions and how they changed remained unknown. To answer this part of the question, we turned to NMR relaxation dispersion experiments. First, it is important to mention that the HDX-MS and NMR are highly complementary. Backbone resonances for many of the surface loops are simply missing from the NMR spectra indicating motions on the ns-µs timescale (Lechtenberg et al., 2010; Fuglestad et al., 2012). The dynamics of these surface loops are revealed by HDX-MS. For motions on the ms timescale, however, relaxation dispersion experiments are the experiment of choice. Relaxation dispersion experiments on thrombin revealed a large number of amino acids throughout the protein were moving on the msec timescale (Figure 6A) (Handley et al., 2017). This was unexpected and unusual for an enzyme where typically such motions are limited to a loop or a few regions at the active site. Binding of the covalent inhibitor, DPhe-Pro-Arg-chloromethylketone (PPACK) to the active site serine dampened many of the motions in thrombin but there remained a group of residues undergoing msec timescale motions that mapped a pathway from ABE1 to the active site (Figures 6B, C) (Handley et al., 2017).

**FIGURE 7:**
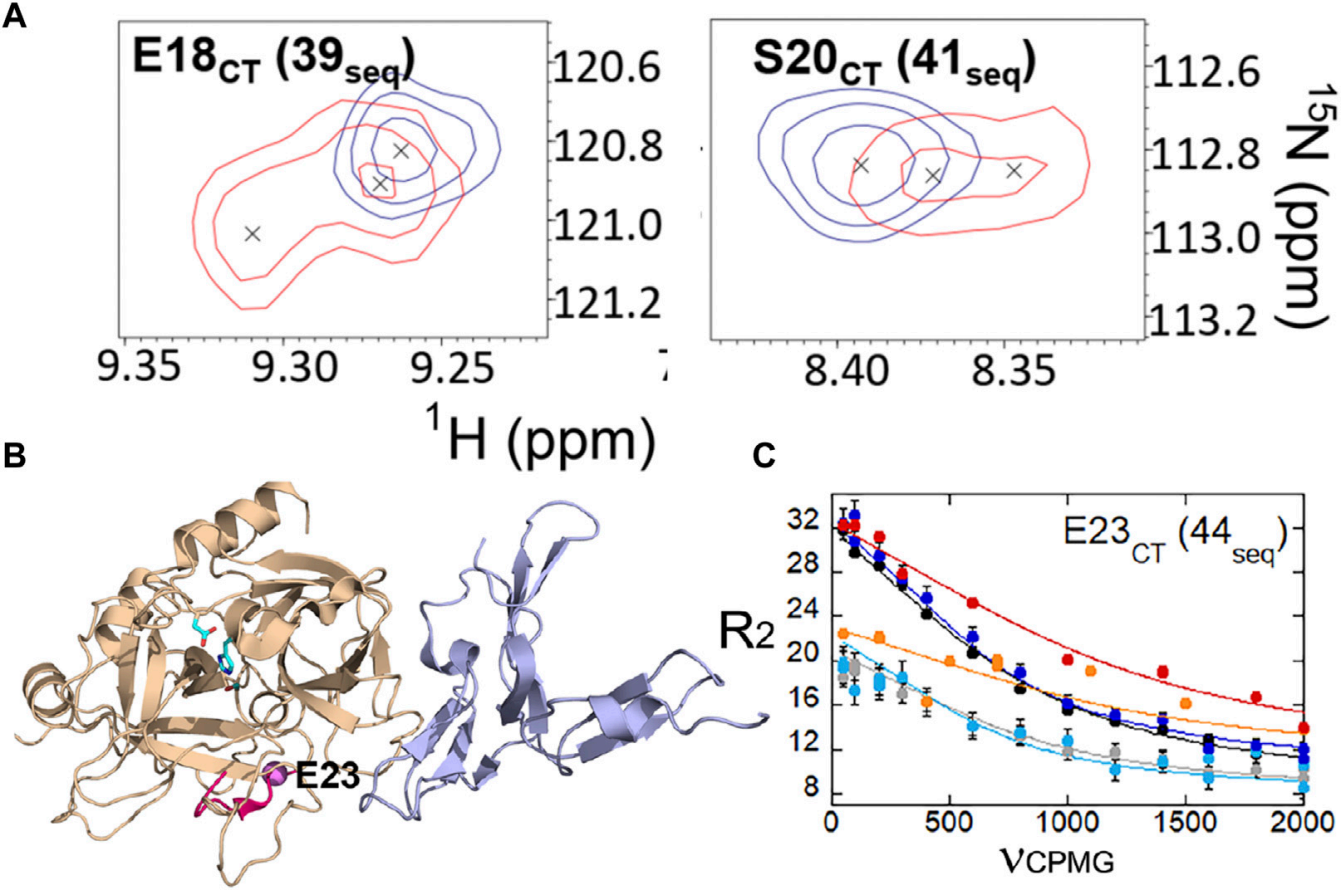
**(A)** Examples of thrombin-TM456 HSQC (red) doublet resonances compared to apo-thrombin (blue) resonances for residues at the N-terminus of the heavy chain. **(B)** Location of residues 16–23 (37-44seq) in the structure of thrombin (wheat) bound to TM456 (light blue) [PDB ID: 1DX5] (hot pink). The resonance corresponding to Glu 23CT (44seq) experienced similar μs-ms dynamics in thrombin-TM456 and apo-thrombin (purple sphere). **(C)** CPMG plot for the resonance corresponding to residue Glu 23 (44seq). The red and orange symbols are from spectra collected on thrombin-TM at 800 MHz and 600 MHz respectively, the blue and cyan curves are from apo-thrombin at 800 MHz and 600 MHz respectively, and the black and grey curves are from PPACK-thrombin at 800 MHz and 600 MHz respectively. These data are taken from Peacock et al. (2021).

**FIGURE 8:**
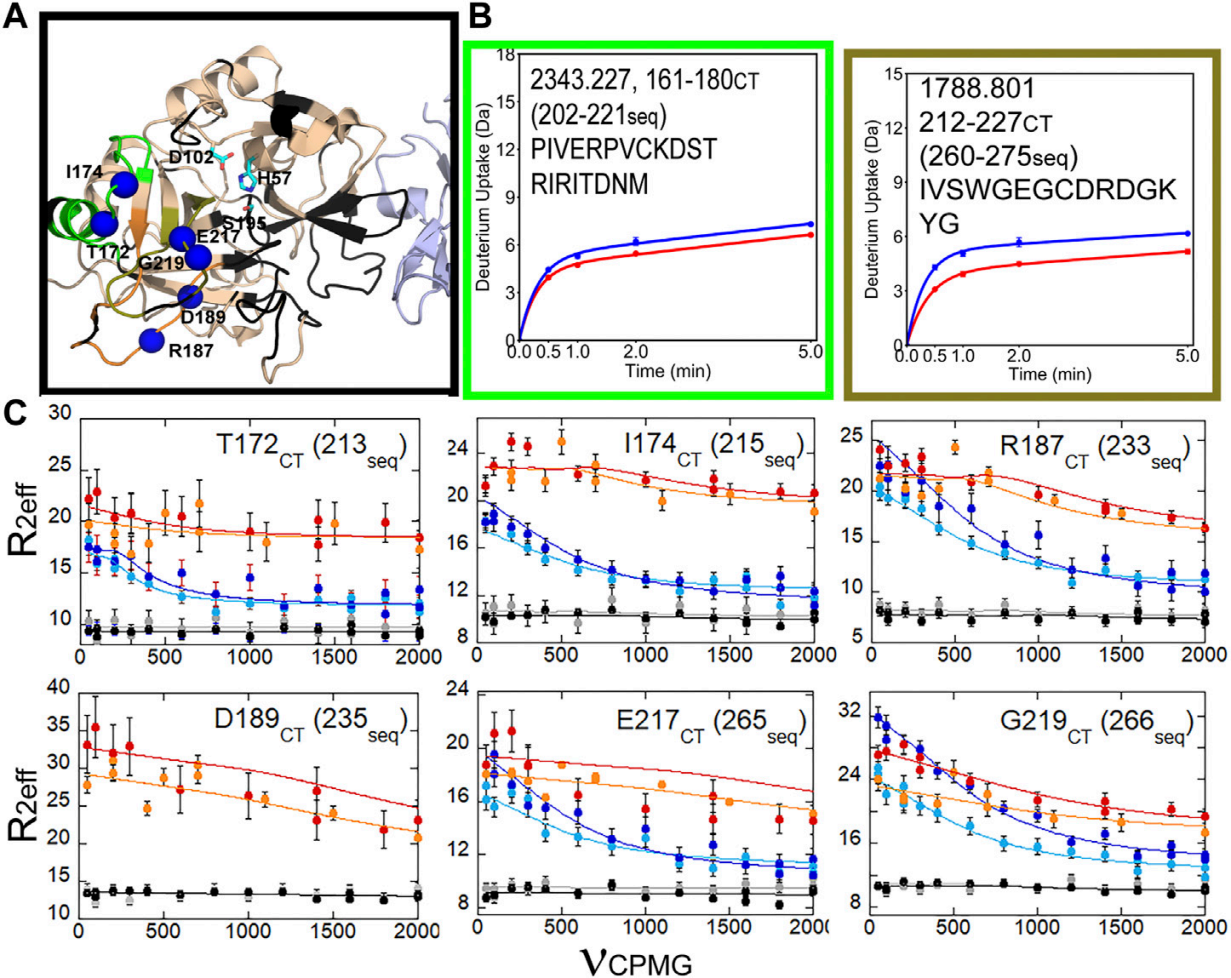
HDX-MS and NMR relaxation dispersion data showing the reduced dynamics in the C-terminal β-barrel of thrombin upon TM binding. **(A)** Structure of thrombin (wheat) bound to TM456 (light blue) [PDB ID: 1DX5]. The residues within the peptides identified in panel **(A)** are colored accordingly. Residues with missing HSQC resonances (black) and residues corresponding to resonances experiencing reduced μs-ms dynamics in thrombin-TM456 compared to apo-thrombin (blue spheres) are shown. **(B)** Deuterium uptake plots for the peptides spanning residues 161–180 (202-221seq), and 212–227 (260-275seq). **(C)** CPMG plots for resonances experiencing reduced μs-ms dynamics around the S1 pocket when TM is bound compared to apo-thrombin (symbols as in Figure 7). These data are taken from Peacock et al. (2021).

We also were able to use relaxation dispersion experiments to measure the motions in the thrombin-TM complex (Peacock et al., 2021). TM-bound thrombin residues in the new N-terminal sequence (IVEGSDAE corresponding to residues 16–23 in the chymotrypsin numbering or residues 37–44 in the sequential numbering) became doublets whereas they were single peaks in the sample of thrombin alone (Figure 7A). Doublets indicate motions that are slow on the NMR timescale. By residue 23 (44 in sequential numbering) the motions were more rapid so that the single peaks were again observed, and relaxation dispersion curves revealed that the motions were similar in thrombin and in the thrombin-TM complex (Figures 7B, C). The other region of the protein that showed doublets was the interface between the two β-barrels that form the two sides of the active site (Peacock et al., 2021). TM binds to the N-terminal b-barrel whereas the substrate binds to the C-terminal β -barrel. Interestingly, TM binding caused increased dynamics in the N-terminal β-barrel where it binds and decreased dynamics in the C-terminal β-barrel on the opposite side of the structure (Figure 8). The C-terminal β-barrel also contains the Na + binding site and the tryptophans that were discussed earlier.

## 6 A synthesis of the different observations of allostery

**FIGURE 9:**
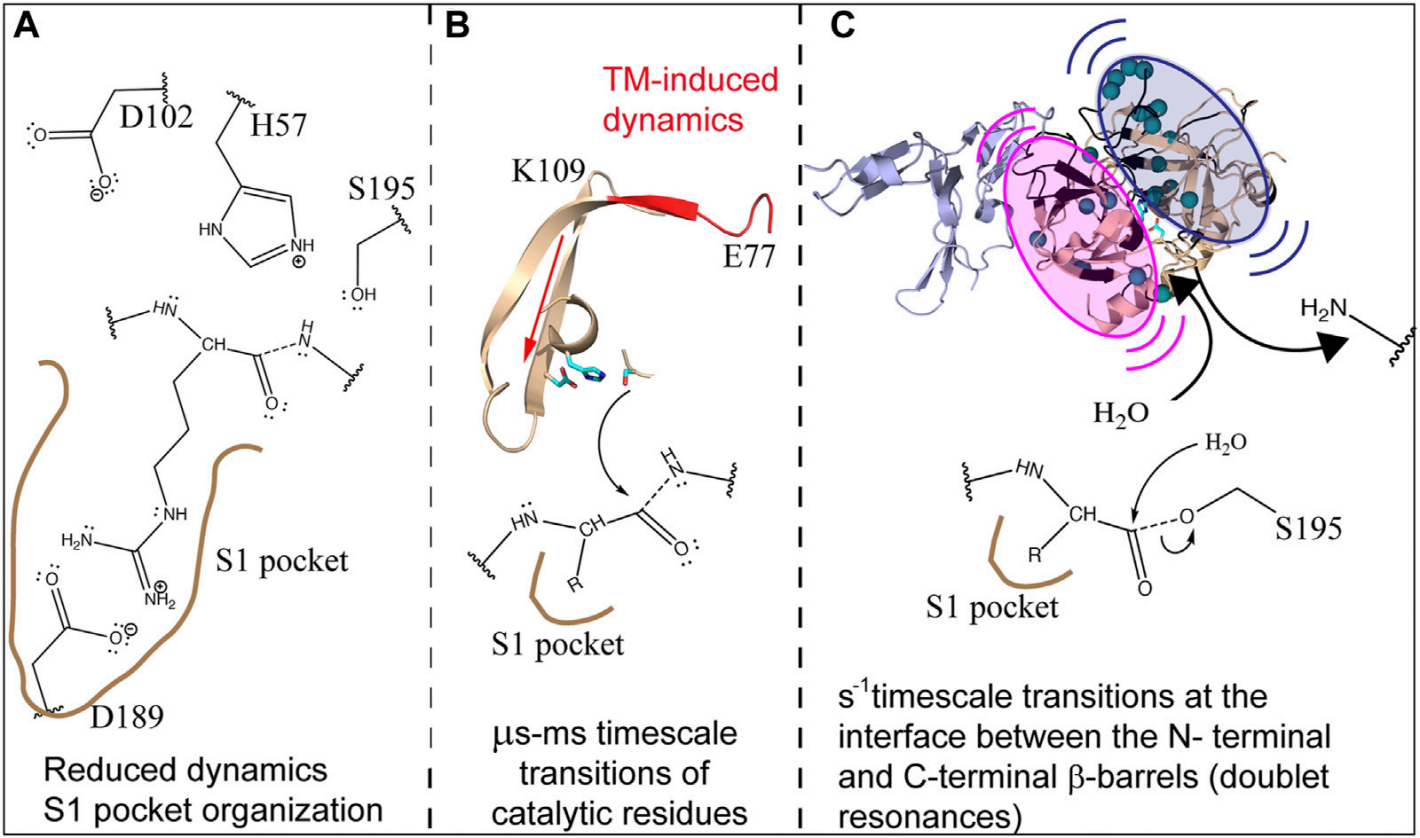
Schematic showing how the dynamics of thrombin are modulated by TM to facilitate cleavage of protein C. **(A)** TM reduces the dynamics and structures the C-terminal β-barrel facilitating substrate association. **(B)** TM drives the motions of the catalytic triad via µs-ms motions. **(C)** TM causes slow motions of the two β-barrels with respect to each other in order to facilitate release of the first product and subsequent entry of H2O into the active site.

The picture that emerges from incorporating lessons from all these different experiments is that ABE1 and the active site are dynamically allosterically coupled. The dynamic allostery involves enthalpy-entropy compensation and from NMR relaxation dispersion experiments we see that whereas TM-binding causes increased dynamics in the N-terminal β-barrel, it causes decreased dynamics throughout the C-terminal β-barrel. In addition, TM causes slow motions that cause doublet signals near the active site. We did not detect the increased dynamics in the N-terminal β-barrel by HDX-MS, probably because the affected regions are β-strands interior to the protein. We did detect the decreased dynamics in the C-terminal β-barrel because the dynamic changes in this β-barrel largely involved loops in which the amide hydrogen bonds would be sensitive to slightly altered structures. The decreased dynamics in the C-terminal β-barrel are consistent with the structuring of the active site for substrate-binding and catalysis. Whereas Na + binding would be required to do this structuring in the absence of TM, Na + binding is not required with TM because TM itself is capable of structuring the active site. It is likely that each of these changes in dynamics plays a role in the overall catalytic mechanism of thrombin. TM-mediated structuring of the substrate binding site would increase the rate of association of protein C with thrombin as has been observed previously (Di Cera et al., 1997). On the other hand, the active site of thrombin needs to move to form the intermediates in the reaction a process that is likely promoted by the motions within the N-terminal β-barrel. It is likely that the slow motions revealed by the doublet peaks at the interface between the two b-barrels facilitate the N-terminal product release and binding of the H2O molecule for hydrolysis of the acylenzyme intermediate (Figure 9).
